# Development of Multilayer Ciprofloxacin Hydrochloride Electrospun Patches for Buccal Drug Delivery

**DOI:** 10.3390/jfb13040170

**Published:** 2022-09-29

**Authors:** Jorge Teno, Maria Pardo-Figuerez, Kelly J. Figueroa-Lopez, Cristina Prieto, Jose M. Lagaron

**Affiliations:** 1 R&D Department, Bioinicia S.L., Calle Algepser 65 nave 3, Paterna, 46980 Valencia, Spain; 2 Novel Materials and Nanotechnology Group, Institute of Agrochemistry and Food Technology (IATA), Spanish Council for Scientific Research (CSIC), Calle Catedrático Agustín Escardino Benlloch 7, Paterna, 46980 Valencia, Spain

**Keywords:** ciprofloxacin hydrochloride, electrospinning, transmucosal patch, biopolyester, antimicrobial

## Abstract

Bacterial infections in the oral cavity can become a serious problem causing pain, sores and swelling for several weeks. This type of infection could be alleviated using mucoadhesive delivery systems, allowing local administration of the antibiotic to inhibit bacterial spreading. This work reports the development of a multilayer antibiotic patch containing ciprofloxacin hydrochloride (CPX)-loaded electrospun fibers for the treatment of such infections. For this, the release kinetics of the CPX-loaded fibers was modulated using different ratios of polyester blends. The selected reservoir layer was analyzed by scanning electron microscopy (SEM), Fourier transform infrared spectroscopy (FTIR), wide angle x-ray scattering (WAXS) and differential scanning calorimetry (DSC). These analyses confirmed the presence and good distribution of the drug in the fibers and that the drug is in an amorphous state within the reservoir layer. To enhance mucoadhesion whilst ensuring drug directionality, the reservoir layer was assembled to a backing and an adhesive layer. This multilayer patch was assessed in terms of in vitro drug release, adhesion and antimicrobial properties. The multilayer strategy showed excellent antimicrobial properties over time and also a strong adhesion patch time in the volunteers for an average of 7 h. These results highlight the capabilities of multilayer electrospun patches as platforms to treat oral infections.

## 1. Introduction

Infections are illnesses caused by certain organisms such as bacteria, fungi, viruses and parasites [1]. Among them, bacterial infections are the most commonly reported, and although some of them may result in minor symptoms that usually resolve themselves, some others may cause serious life-threatening conditions, such as pneumonia or sepsis [2,3,4]. Bacterial infections can also be a serious problem in the oral cavity, with a particular worst-case scenario for immuno-suppressed patients, where the microorganisms present in the mouth can cause ulcers and wounds that can last for weeks [5,6,7,8,9,10]. These infections are commonly treated with formulations based on gels, sprays, tablets and mucoadhesive systems, with the latter presenting the most promising effects [6,7,11]. Mucoadhesive delivery systems present numerous advantages such as easy administration, sustained delivery to prevent bacteria spreading, rapid onset of action and avoidance of first-pass effect for certain drug types [1,12,13]. Additionally, these systems are often preferred to conventional dosage forms for the patients since they protect the site of action and are more comfortable to use [1,5].

Although mucoadhesive drug delivery presents many advantages, the vast majority of drugs, such as antibiotics, are most popularly administered through oral delivery and subsequent gastrointestinal tract absorption [1,14]. However, this route of delivery is quite limited, not only for the reported side effects but also for the low solubility and permeability of commonly used antibiotics, which are mainly classified as class II (low solubility) or IV (low solubility and low permeability) according to the biopharmaceutical classification system (BCS) [2,15]. In an attempt to increase the bioavailability of such active ingredients, the technique of electrospinning has been frequently used. This technique can be used to generate solid dispersions of an API within a polymer matrix, generating solid pharmaceutical formulations that can adhere to the mucosa membrane. Electrospinning is based on the application of a high electric field to a polymeric solution to generate ultrathin fibrous structures. The rapid evaporation rate of the solvent during the process leads to rapid entrapment of the drug within the polymeric matrix, leaving little possibility for the API to reorganize itself and crystalize [16,17]. Furthermore, the resultant fibrous materials have a high specific area, tunable pore size and controlled mechanical properties, making them attractive in drug delivery applications [18].

Different electrospinning methods such as blending [19], coaxial [20] or emulsion electrospinning [21] are used to efficiently encapsulate active ingredients. It is known that API release kinetics can be tuned with the right selection of the polymer matrix, the morphology, as well as the compatibility of the drug–polymer matrix [19,22]. With an appropriate selection of these parameters, the electrospun fibers can provide a controlled release of antibiotics at the local site, thus actively preventing infection.

Herein we present the development of a multilayer system for the release of ciprofloxacin hydrochloride (CPX) by means of blend electrospinning. CPX is an antibiotic belonging to the fluoroquinolones family, with broad antibacterial activity against Gram-negative and Gram-positive bacteria [7]. Such antimicrobial effects make CPX attractive for treating infections in the urinary tract, skin [23,24], sexually transmitted diseases such as HIV [25] and oral diseases [5,6,7]. Being considered a class IV drug by the BCS, CPX has limited water solubility at physiological pH as well as a heavy first-pass effect, which makes it an ideal candidate to be administered in a mucoadhesive drug delivery system [7,15].

Some studies have presented the vehiculation of CPX in electrospun fibers. In a study presented by Yarin et al. (2016), the release of CPX was modulated by electrospinning a blend of Poly(methyl methacrylate) (PMMA) with hydrophilic polymers [19]. The different hydrophilic polymers used in the blend (chitosan, polyethylene oxide (PEO) or polyvinyl alcohol (PVA)) dramatically altered the CPX release profiles, with chitosan revealing a slow, gradual release and PEO delivering a burst effect, while PVA yielded a two-stage release. Similarly, Moydeen et al. (2018) utilized core–shell nanofibers of PVA/dextran prepared by emulsion electrospinning to encapsulate CPX. In vitro drug release studies showed that the PVA/Dextran nanofibers could modulate a sustained drug release of CPX, and this could be further tuned by altering the concentration of dextran in the mixture [26]. Another study reported by Wu et al. (2020) presented the delivery of CPX with polymeric blends, in which the second polymeric component modulated the release of CPX [27]. These studies efficiently analyzed the release kinetics of CPX; however, they were based on monolayer systems and did not specify the end application, making it difficult to correlate the release kinetics data with the application time of the delivery system at the site of action. 

In the case of mucoadhesive systems for drug delivery, a study based on a buccal cast film for CPX delivery was carried out by Kanango et al. [7]. In this case, the study reported a release of CPX for up to 6 h, with a mucoadhesion time of approximately 211 min. A similar study based on the oral mucosa release of metronidazole reported a release time of up to 7 h, with an average mucoadhesion of approximately 2 h [10]. Mucoadhesive electrospun patches for the release of clobetasol have also been reported in the literature, where authors reported a clobetasol release close to 100% after 360 min, along with a patch residence time of approximately 120 min [28]. According to these studies, it appears that the release kinetics of the API are often designed for mucoadhesive platforms to match the relatively short average mucoadhesion time of the patch. 

This work presents a multilayer CPX drug delivery system developed to simultaneously adhere to the mucosa, support drug unidirectionality and treat antimicrobial infections in the oral cavity. We previously reported a multilayer approach based on carvedilol (CVD)-loaded fibers, in which a trilayer system modulated the adhesion and release of CVD [17]. For the work presented here, a reservoir layer containing CPX, a mucoadhesive layer (ADH) and a backing layer (BL) were assembled independently to obtain a sustained drug delivery system. Firstly, a blending strategy using two polymers, poly-ε-caprolactone (PCL) and poly(lactic acid) (PLA), was used to finely tune the release of CPX. The most optimum formulation in terms of release rate was chosen as the reservoir layer and was characterized by scanning electron microscope (SEM), attenuated total reflectance Fourier transformed infrared spectroscopy (ATR-FTIR), wide angle x-ray scattering (WAXS) and differential scanning calorimetry (DSC). The reservoir layer was then embedded into a sandwich-like structure within the adhesive and the backing layer. Lastly, the release kinetics of the multilayer, as well as its antimicrobial and adhesive characteristics, were analyzed to assess the effectiveness of the antibiotic multilayer system. 

## 2. Materials and Methods

### 2.1. Materials

Ciprofloxacin hydrochloride monohydrate (CPX) was obtained from Uquifa (Castellbisbal, Spain). Poly-ε-caprolactone (PCL, Mw 80000 Da) was supplied by Perstorp Caprolactones Ltd (Warrington, United Kingdom), and poly(lactic acid) (PLA viscosity midpoint 2.0 dL/g) was supplied by Natureworks® (Naarden, Netherlands). For the mucoadhesive layer, polyethylene oxide (PEO, Mw 200000 Da) was purchased from the Dow Chemical Company (Montomeryville, PA, USA), and Eudragit RS100 (PAC, copolymer of ethylacrylate, methyl methacrylate and trimethylammonioethyl meth-acrylate chloride) was purchased from Evonik Industries (Essen, Germany). Chloroform (ACS reagent, ≥99.8%) was supplied by Sigma-Aldrich (Madrid, Spain), whilst methanol (≥99%) was supplied by Panreac Química S.L.U. (Castellar del Vallès, Barcelona). The solvent 1,1,1,3,3,3-hexafluoro-2-propanol (HFIP) was supplied by Fluorochem (Hadfield, United Kingdom) and N,N,-dimethylformamide (DMF, ≥99.8%) was purchased from VWR Chemicals (Leuven, Belgium). All the polymers and reagents were used as received without further purification.

### 2.2. Solution Preparation 

Solutions were prepared by dissolving each polymer in its corresponding solvent, as indicated in Table 1. A concentration of 8 wt.% was used for the different reservoir layers and the backing layer (BL), however the concentration was 13 wt.% for the mucoadhesive layer (ADH). In some cases, the surface tension was measured using a DynoTester tensiometer from Krüss GmbH (Hamburg, Germany). In all cases, an amount of 2 wt.% in a solution of CPX was added, so the loading of CPX in the materials was 20%.

### 2.3. Electrospinning

The electrospinning process was carried out in a high throughput Fluidnatek^TM^ LE-100 equipped with a multi-emitter injector from Bioinicia S.L. (Valencia, Spain) and coupled with an air-conditioned unit system. Table 2 shows the electrospinning parameters used for each solution. The environmental conditions were kept at 25 °C and 30% relative humidity (RH) for all the electrospinning processes.

**Figure 1:**
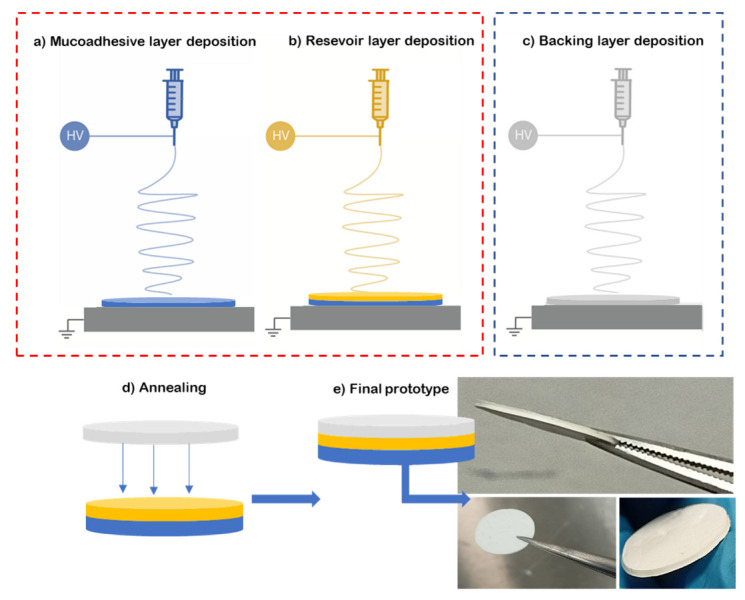
Diagram of the multilayer assembling. (**a**) Electrospinning of adhesive layer (blue, ADH); (**b**) electrospinning of reservoir layer over the adhesive layer (yellow); (**c**) backing layer electrospun separately from the previous layers (grey, BL); (**d**) lamination by low-temperature annealing of the backing layer against the bilayer patch; (**e**) resultant prototype of the multilayer approach.

To prepare the multilayer patch, a three-layer structure was prepared. For this, the mucoadhesive layer and reservoir layer were electrospun in a “layer-by-layer” approach, where one layer was electrospun on top of the other one (see Figure 1a,b). Then, a hydrophobic backing layer (BL) made of electrospun PCL fibers was electrospun separately (Figure 1c) and laminated to the previously prepared bilayer patch. The lamination was carried out using a hot press (Carver 4122, Wabash, IN, USA), programmed at 50 °C for 10 s without pressure at the top plate facing the backing layer, meaning that no temperature on the bottom plate was applied in the adhesive layer zone to assemble the whole multilayer patch (Figure 1d,e). The backing layer of the patch is designed to act as an impermeable layer due to the coalescence of the fibers to promote drug unidirectionality towards the mucosa, and so it is pivotal to the structure of the patch. The surface density was optimized at 20 g/m^2^ for the backing layer, 60 g/m^2^ for the reservoir layer and 100 g/m^2^ for the adhesive layer.

### 2.4. Fiber Morphology (SEM)

Fiber morphology was analyzed by scanning electron microscopy (SEM) using a Phenom XL G2 Desktop microscope (Thermo Fisher Scientific, Waltham, MA, USA) with an electron beam acceleration of 5 kV. Microanalysis was performed by energy-dispersive X-ray spectroscopy with a Hitachi S-4800 FE-SEM (EDS, Hitachi High Technologies Corp., Hitachi, Japan). Average fiber diameter based on at least 100 fibers was determined using Phenom ProSuite Software on SEM images. 

### 2.5. Fourier Transform Infrared (FTIR)

The placebo and CPX electrospun blends were evaluated with Fourier transformed infrared spectroscopy and measured with a Bruker Tensor 37 FT-IR Spectrometer (Bruker, Ettlingen, Germany) coupled with the attenuated total reflectance (ATR) sampling accessory Golden Gate (Specac Ltd., Orpington, UK). Spectra were collected from an average of 64 scans in the range of 400–4000 cm^−1^, with a resolution of 4 cm^−1^.

### 2.6. Differential Scanning Calorimetry (DSC)

The electrospun fibers were studied by differential scanning calorimetry (DSC) on a DSC-8000 analyzer from PerkinElmer, Inc. (Waltham, MA, USA), equipped with a cooling accessory Intracooler 2 also from PerkinElmer, Inc. Approximately 3 mg of each sample were placed in standard aluminum pans and heated from 0 to 300 °C and at a rate of 10 °C/min using a nitrogen flow of 20 mL/min as the sweeping gas.

### 2.7. Wide-Angle X-ray Scattering (WAXS)

Samples were scanned by wide-angle X-ray scattering at room temperature in reflection mode using a Bruker AXS D4 Endeavor diffractometer (Bruker, Ettlingen, Germany). The samples were analyzed using incident Cu K-alpha radiation (Cu Kα = 1.54 Å) while the generator was set up at 40 kV and 40 mA. The data were collected over a range of scattering angles (2Θ) in the 5–40° range.

### 2.8. In Vitro Drug Release 

Drug release was evaluated using a UV-spectrophotometer DINKO UV4000 (Barcelona, Spain). The release of the CPX-loaded monolayer and multilayer patches (2.19 cm^2^) into an aqueous phosphate-buffered solution (pH 6.8) medium was monitored by measuring the absorbance at 270 nm at predetermined times whilst stirring at 100 rpm at 37 °C. The reading was compared against a calibration curve produced using standard samples of 1–8 µg/mL of CPX in buffer solution (pH 6.8). The placebo patches were also measured to ensure that the polymeric matrix did not interfere with the CPX reading. A total of 1 mL aliquot of the solution was withdrawn at each time point for UV–vis analysis, and an equal volume of fresh buffer solution was added to maintain the sink conditions. The patches were attached to supports and lowered into the solution media. In the case of the multilayer approach, the patch was placed with the adhesive layer facing toward the buffer solution to somehow mimic the initial contact with the mucosa. Since the purpose of the study was to generate a patch for oral mucosa delivery, a relatively low release time (8 h) was intended, taking into consideration that the mucoadhesion of the patch may, in principle, last for a few hours for patient comfortability. The release experiments and the standard curves were carried out in triplicate, and the cumulative release rate was reported as mean ± S.D. 

### 2.9. Determination of Experimental Loading Capacity

To determine the experimental loading of CPX within the fibers, the CPX-loaded monolayers (2.19 cm^2^), weighing approximately 2 mg, were dissolved in 60 mL of HFIP to fully dissolve both CPX and the polymeric matrix. The resulting solution was stirred at 100 rpm for an hour and then analyzed by UV-spectrophotometer DINKO UV4000 (Barcelona, Spain). The reading was compared against a calibration curve produced using standard samples of 1–8 µg/mL of CPX in HFIP. The absorbances at 270 nm were recorded, and the experimental CPX loading (%) in the fibers was calculated using Equation (1):

(1) $$\mathrm{CPX}\;\mathrm{Loading}\;\left(\%\right)=\frac{m_{d}}{m_{p}}\times 100,$$

where m_d_ is the mass of the drug obtained experimentally as described above, and m_p_ is the total mass of the sample analyzed. Similarly, the process yield was calculated as follows:

(2) $$\mathrm{Process}\;\mathrm{yield}\left(\%\right)=\frac{\mathrm{experimental}\;L}{\mathrm{theoretical}\;L}\times 100$$

where process yield is calculated as the ratio between the theoretical and experimental loading of the drug in the solution and the patch, respectively.

### 2.10. Kinetic Model of In Vitro Drug Release

To analyze the kinetics of the in vitro drug release behavior of the fibers, the semi-empirical mathematical model of Korsmeyer–Peppas was applied as follows:

(3) $$Q=kt^{n}$$

where *Q* is the amount of drug released in time *t*, *K* is the Korsmeyer–Peppas release rate constant and *n* is the release exponent, which depends on the type of drug polydispersity, geometry and transport. Depending on the release exponent, diffusional release mechanisms were classified as; *n* < 0.5 pseudo-Fickian diffusional behavior, *n* = 0.5 Fickian diffusion, 0.5 < *n* < 1 non-Fickian diffusion, *n* = 1 case II transport (zero-order release) and *n* > 1 super case II transport [29].

### 2.11. In Vitro Patch Residence Time Study 

For the in vitro adhesion studies, a qualitative study was carried out in which 1 mL of aqueous phosphate-buffered solution (pH 6.8) was initially deposited on 60 × 15 mm size Petri dishes. Thereafter, the electrospun multilayer of 2.19 cm^2^ size (with the adhesive layer facing the Petri dish) was applied on the wet plastic surface of the Petri dishes by pressing firmly on top of the patch (backing layer side) for 5 s. The test was carried out at 37 °C. A visual inspection of the adhesion of the patch to the surface of the Petri dish was carried out at 30 min and 90 min.

### 2.12. Mucosa In Vivo Patch Residence Time 

In order to determine the overall in vivo adhesion time and patch acceptability, multilayer patches were tested by six healthy human volunteers (three males and three females) ages between 25–50 years old. Similarly to other studies [30,31,32], the volunteers were asked to apply the multilayer patches in the gingiva region for 5 s with applied pressure. The residence time of the patch was defined as the time interval between the application of the patch and the time point where it moved, detached or could not be felt any more at the site of application. During the test period, volunteers were allowed to drink, eat and speak.

### 2.13. Antimicrobial Activity

Staphylococcus aureus (*S. aureus*) CECT240 (ATCC 6538p) and Escherichia coli (*E. coli*) CECT434 (ATCC 25922) strains were obtained from the Spanish Type Culture Collection (CECT, Valencia, Spain). The bacteria were thawed at 37 °C under agitation at 180 rpm for 24 h and were then diluted in a 1:1000 proportion in a Mueller–Hinton broth until obtaining a concentration of 3–5×10^5^ colony-forming unit (CFU)/mL [33].

The antimicrobial activity of the multilayer patch was studied by the disc diffusion method, following the protocols of the Clinical and Laboratory Standards Institute (CLSI) with some modifications [33,34]. Patches of 2.19 cm^2^ with (CPX multilayer patch, approximately 2.6 mg of CPX) and without CPX (placebo multilayer patch, control) were deposited onto the Mueller–Hinton solid agar surface previously inoculated with 100 μL of *S. aureus* and *E. coli* (3–5 × 10^5^ CFU/mL). The samples were analyzed in triplicate and incubated at 37 °C for 24 h. Inhibition diameters were measured after incubation, and the results are presented as the mean ± S.D.

The antimicrobial performance of the patches was also determined based on the guidelines of the macro-dilution protocol, as described in the Methods for Dilution Antimicrobial Susceptibility Tests for Bacteria that Grow Aerobically [35]. For this, patches with and without CPX (placebo) with sizes of 2.19 cm^2^ were placed in a tube previously inoculated with *S. aureus* and *E. coli* (3–5 × 10^5^ CFU/mL). The tubes were incubated at 37 °C, and aliquots were taken at 1, 3, 6 and 8 h. Thereafter, the aliquots were 10-fold serially diluted and incubated at 37 °C for 24 h to quantify the number of viable bacteria by a conventional plate count. Each test was conducted in triplicate. The results are presented as bacterial density expressed as Log CFU/mL. 

## 3. Results and Discussion

### 3.1. Reservoir Layer

#### 3.1.1. Effect of Polymer Blends on Release Kinetics of CPX

**Figure 2:**
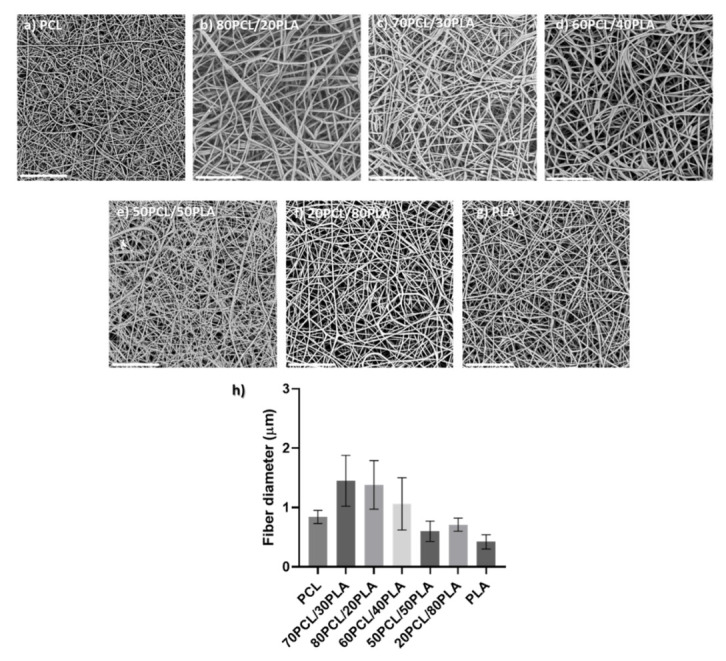
(**a**–**g**) Scanning electron microscopy (SEM) micrographs of the different blends prepared by electrospinning containing CPX (20%) and (**h**) their average fiber diameter (µm). Scale bar= 30 µm, Magnification (×2500) for all the samples.

Blends of PCL and PLA containing CPX were electrospun to generate different reservoir layers in an attempt to modulate the release kinetics of CPX. Although the electrospinning parameters were modified slightly for each blend, all of them had a similar smooth fiber morphology and a fiber diameter between 0.5 to 1.5 µm (see Figure 2). 

Thereafter, the loading of CPX was then measured to ensure the presence of CPX in the matrices. Table 3 gathers the results obtained on the experimental CPX loadings for the different electrospun fibers. All the monolayers presented experimental loadings close to the theoretical ones (20%), which led to close to 100% process yield, indicating the suitability of electrospinning for the processing of APIs to generate efficient solid pharmaceutical forms. 

**Figure 3:**
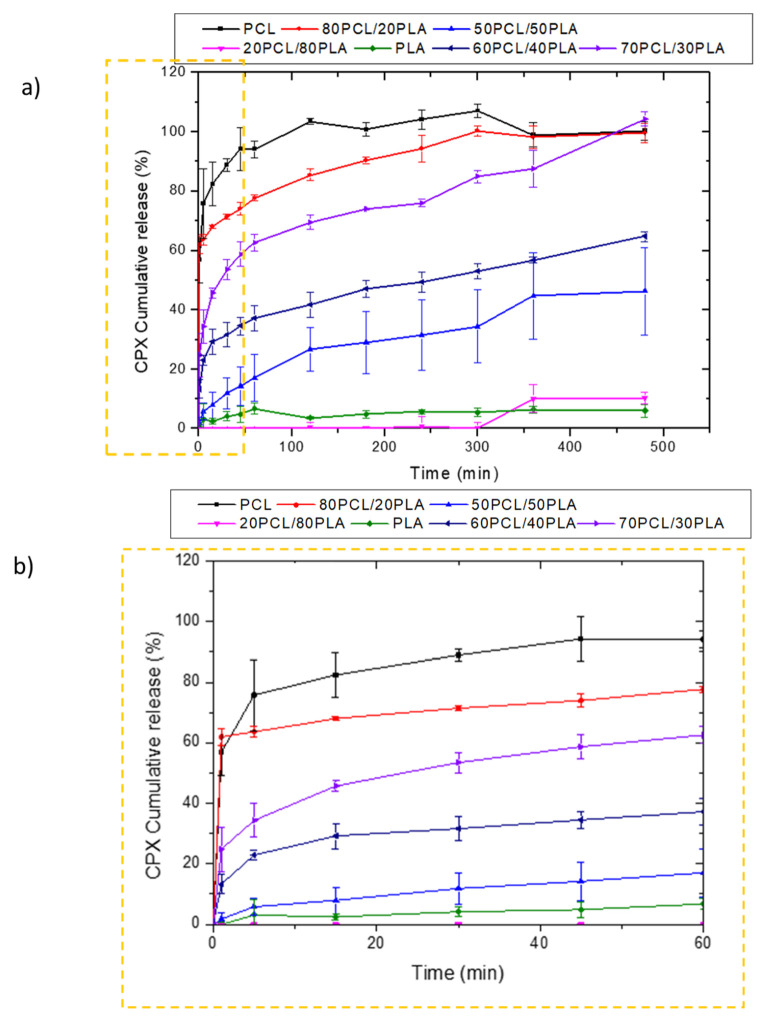
Release profiles of CPX from electrospun PCL/PLA blends. (**a**) Blends PCL, 80PCL/20PLA, 70PCL/30PLA, 60PCL/40PLA, 50PCL/50PLA, 20PCL/80PLA and PLA; (**b**) zoomed zone of the first hour to analyze the initial burst effect. The graphs show the mean ± S.D. (*n* = 3).

Figure 3 shows the release behavior of CPX-loaded electrospun fibers prepared with PCL, 80PCL/20PLA, 70PCL/30PLA, 60PCL/40PLA, 50PCL/50PLA, 20PCL/80PLA and PLA. As can be observed in Figure 3a, the pure PLA and PCL encapsulating materials presented antagonist behavior. The release of CPX in the electrospun PLA fibers showed very slow release over time, whilst PCL presented a burst release with complete release after 2 h. In the case of PCL, the well-known high drug permeability of the PCL polymer was in good agreement with previous studies [21,36,37]. On the other hand, the slow release of PLA, also reported previously, was attributed to its higher intermolecular cohesion and rigidity [36]. Such differing release rates could be used to obtain a tailor-made delivery by blending the two materials. When blending both biopolymers, it was observed that the blend 20PCL/80PLA, which contained the highest amount of PLA, exhibited similar behavior to neat PLA fibers. On the other hand, blends with a higher PCL content (80PCL/20PLA) showed a similar behavior to PCL but with somewhat arrested release compared to pure PCL (Figure 3a). Surprisingly, when the 50PCL/50PLA was analyzed, a limited burst with slower drug release than expected was observed, which was more similar to the behavior of the neat PLA, hence resulting in a negative deviation from the simple rule of mixtures.

Other intermediate blends, i.e., 70PCL/30PLA and 60PCL/40PLA, were also prepared to further modulate drug release (Figure 3a,b). Interestingly, when the blend of 60PCL/40PLA was analyzed, a burst followed by suspended drug released behavior was observed (Figure 3b), similar to what was observed for the 50PCL/50PLA blend. In this case, the CPX was not released entirely during the time of the study. The 70PCL/30PLA blend showed more sustained release than the blend of 80PCL/20PLA, reaching approximately 100% of drug release after 8 h. For the delivery of antibiotics at the local site of action, an initial burst can be ideal for inhibiting bacterial growth before proliferation. Thereafter, more arrested release is convenient to prevent bacteria proliferation of the organisms that resisted the initial burst [37]. The blend 70PCL/30PLA was selected as the reservoir layer to be employed for the design of the multilayer patch. 

Polymer blending was previously used as an important strategy to tailor drug release [37,38,39,40]. Basar et al. (2017) reported the release modulation of ketoprofen by preparing blends of PCL and gelatin [21]. Malagón et al. (2015) used blends of PCL and PLA to analyze the different release kinetics of amoxicillin. In agreement with this study, rapid release occurred for the electrospun pure PCL-loaded amoxicillin, whereas slow release was reflected for the pure PLA-loaded fibers. Interestingly, the release in the blends was not seen to follow the rule of mixtures either, and the effect was attributed to better compatibility of the antibiotic with the PLA [37]. Similarly, a study by Chou et al. [41] reported an increase in Young’s modulus of blended polymer fibers made of PLGA and PCL when PLGA composition increased from 0% to 100%. However, this trend varied significantly and did not follow the rule of mixtures either when the polymer blend fibers were loaded with a 15 wt.% of tenofovir (TFV). 

In our study, a clear difference can be seen in the release of CPX when PCL becomes the predominant phase in the blend. However, intermediate blends such as 50PCL/50PLA and 60PCL/40PLA deviate very strongly from the rule of mixtures. Thus, it is visible that the blends containing a major ratio of PLA undergo a release arrest of CPX, hence suggesting better compatibility of CPX with PLA. There are, in fact, many potential parameters involved in the release of drugs, such as the morphology of the nanofibers, the crystalline morphology at the mesoscale of the blend components, the physical state of the drug and also the different drug–polymer affinities that may ultimately impact its release [37,41].

#### 3.1.2. Characterization of the Selected Reservoir Layer

**Figure 4:**
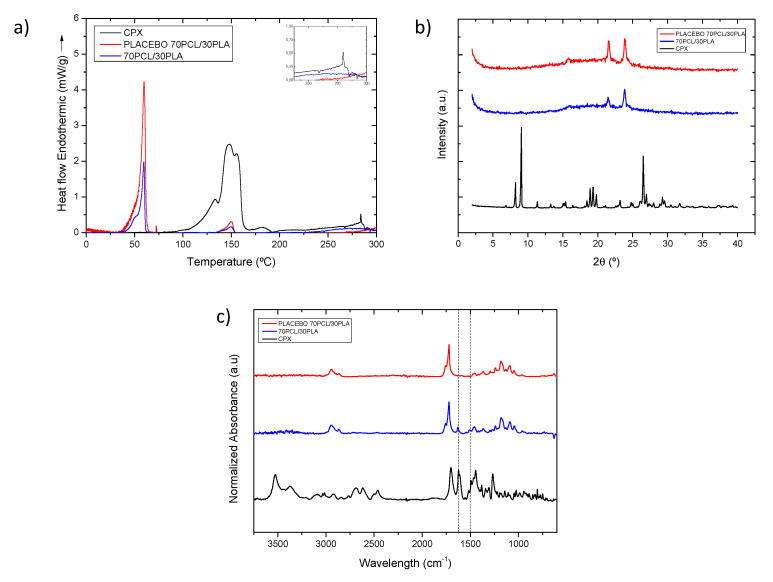
(**a**) Normalized to the polymeric content DSC thermograms and inset with the zoomed out area in the temperature range 250–300 °C, (**b**) WAXS diffractogram and (**c**) normalized intensity ATR-FTIR spectra of samples of CPX powder (black line), placebo electrospun blend (red line) and electrospun CPX containing blend (blue line). The dashed vertical lines indicate the CPX characteristic peaks at 1622 and 1492 cm^−1^.

The selected 70PCL/30PLA electrospun material was characterized further to obtain information regarding the physical state of CPX and the polymeric blend. Figure 4a shows the DSC thermograms of the selected reservoir layer (70PCL/30PLA), as well as its placebo counterpart (without CPX) and pure CPX. The thermogram of the CPX showed a broad endothermic peak around 150 °C when it ran up to 300 °C, ascribed to dehydration and the loss of moisture corresponding to water molecules that bound directly to the cationic ciprofloxacin, which dehydrates between 140–160 °C [42,43,44,45,46,47]. Studies in the literature reported that ciprofloxacin hydrochloride monohydrate sequentially melts and degrades in the range of 310–340 °C [45,47], whereas others reported that anhydrous ciprofloxacin melted around 270–280 °C [24]. In this thermogram, a small endothermic peak was present at around 285 °C, suggesting that there may be a part of anhydrous CPX present in the sample, perhaps resultant of the initial water loss. The peaks beyond 300 °C were not analyzed because the blend components would have degraded.

For the thermogram of the placebo electrospun blend (70PCL/30PLA), a clear thermal endothermic peak at 59.78 °C, corresponding to the melting point of PCL crystals, was found. Additionally, a small endothermic feature could be seen at 150.2 °C, which was attributed to the melting point of PLA. As per the CPX-containing blend, the signal of PCL and PLA can also be discerned at 59.50 °C and 149.73 °C, respectively. In this case, both peaks, after normalization of the endotherm for the number of polymers present, were smaller in area when compared to the placebo blend. This suggests that API impedes the development of crystallinity in both polymeric phases.

As per the physical state of the drug in the polymeric matrix, this could not be analyzed via DSC since, on the one hand, the degradation of the polymers could influence the endotherm signal beyond 300 °C, and the PCL (T_m_ ca. 60 °C) and PLA (T_m_ ca. 150 °C) melting before the CPX (T_m_ ca. 300–340 °C) could dissolve the CPX crystals [48]. Thus, to assess the physical state of the drug, WAXS analysis was carried out instead (Figure 4b). 

The diffractograms in Figure 4b clearly show the X-ray diffraction patterns of the neat CPX powder, exhibiting a clear crystalline nature presented by various smaller peaks and three main sharp ones at around 2-Theta = 8.2°, 9.1° and 26.5°, in agreement with previous studies [49]. For the blend containing CPX and its placebo, the only crystalline peaks that were unambiguously discerned were those associated with the crystals of the PCL in the polyester electrospun blend (70PCL/30PLA), also in agreement with other studies [50]. Interestingly, the placebo spectra show sharper peaks than the blend containing CPX, again suggesting a higher degree of crystallinity for the blend without API. Nevertheless, the lack of CPX representative peaks in the CPX-containing blend confirmed that the CPX was most likely present in an amorphous solid form of the API within the fibers, hindering the arrangement of macromolecular chains in the fibers.

Figure 4c shows a comparison of the ATR-FTIR spectra of the CPX powder, the placebo blend and the CPX-loaded blend. The placebo and CPX-loaded blend showed the main characteristic bands of PCL at 2946, 2870, and 2821 cm^−1^ assigned to CH_2_ vibrations, as well as C=O and C-O-C stretching, which were seen at 1720 cm^−1^, 1238 cm^−1^ and 1161 cm^−1^, respectively. In addition, the main characteristic peak of PLA at 1080 cm^−1^ associated with the C-O-C stretching was also observed [51]. The CPX powder presented characteristic peaks at 1622 and 1492 cm^−1^ (dashed lines in Figure 4c), associated with C=O and C-H stretching, respectively [52,53]. Regarding the blend 70PCL/30PLA, the more intense characteristic peaks of CPX can be clearly seen in the blend, as suggested in the dashed lines. Therefore, the ATR-FTIR results confirm the presence of CPX within the polymer blend.

### 3.2. CPX Multilayer Patch 

#### 3.2.1. Patch Morphology Characterization 

**Figure 5:**
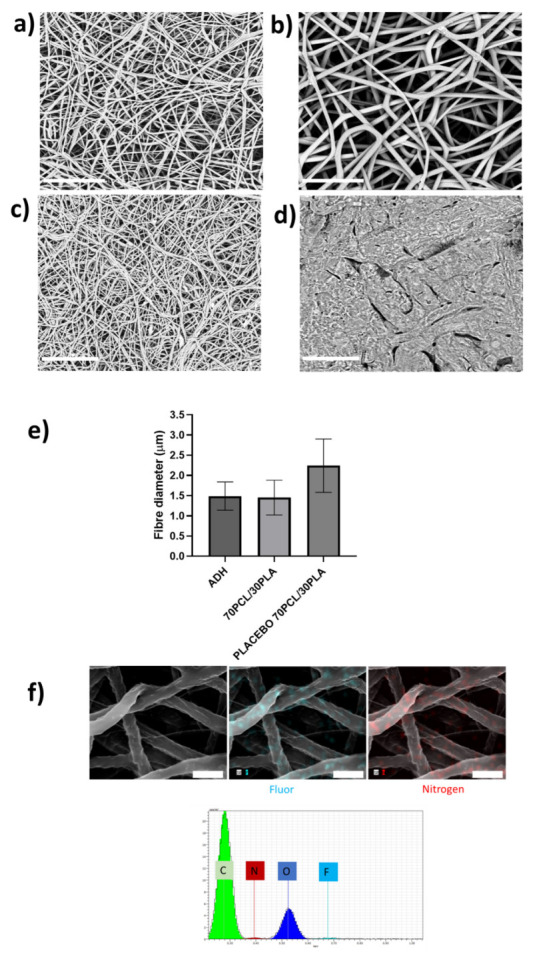
Scanning electron microscopy (SEM) micrographs. (**a**) Blend 70PCL/30PLA containing CPX, (**b**) placebo blend 70PCL/30PLA, (**c**) adhesive layer ADH and (**d**) backing layer after the annealing process. Scale bar = 30 µm, magnification (×2500). (**e**) Fiber diameter of the adhesive layer (ADH), the reservoir layer 70PCL/30PLA and the placebo layer (placebo 70PCL/30PLA). (**f**) Energy-dispersive elemental mapping micrographs of the characteristic elements of CPX. EDS microanalysis spectrum with the main components found is also included (Scale bar = 4 µm).

As described in Figure 1, the developed patch consisted of a backing layer based on annealed occlusive electrospun PCL fibers to direct the drug delivery towards the mucosa and a mucoadhesive layer to promote patch adhesion to the oral cavity. The different layers of the CPX multilayer patch made with the 70PCL/30PLA reservoir layer were analyzed under SEM (see Figure 5). The electrospun reservoir layer with and without CPX had a fibrous morphology randomly distributed; however, subtle changes in fiber size were observed when adding CPX (see Figure 5a,b,e). The placebo 70PCL/30PLA fiber size was 2.24 ± 0.7 µm, and for 70PCL/30PLA it was 1.45 ± 0.43 µm. The addition of active ingredients into a polymer solution often results in changes in the solution properties, leading to a change in processability and final fiber morphology. The addition of CPX resulted in a slight increase in the surface tension of the solution (placebo 70PCL/30PLA= 35.7 mN/m; 70PCL/30PLA = 38.4 mN/m), which could be responsible for the decrease in fiber diameter. In terms of processability, both formulas were electrospun under the same conditions, concluding that the addition of CPX did affect fiber diameter but did not affect the productivity or processability of the materials. 

Figure 5c,d show the morphology of the adhesive layer (ADH) and the backing layer, respectively. The backing layer exhibited fused PCL fibers due to the annealing step (Figure 5d), whilst the adhesive layer showed a fine random fibrous morphology (Figure 5c). Lastly, Figure 5f shows the EDS mapping of Nitrogen and Fluor elements and the EDS spectrum of the electrospun CPX-containing fiber layer. The spectrum showed the presence of Carbon (C) and Oxygen (O), characteristic elements of the PCL, PLA and CPX, but also Nitrogen and Fluor were detected, elements characteristic of the CPX. The EDS mapping analysis of the F and N elements confirmed the presence and good dispersion of the CPX in the fibers.

#### 3.2.2. Effect of the Multilayer Structure in Drug Release

**Figure 6:**
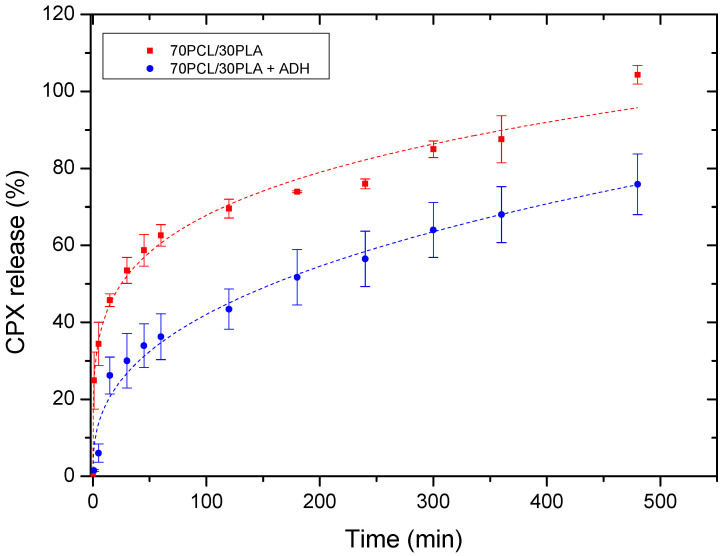
Drug release data comparing the monolayer reservoir layer (70PCL/30PLA) and the multilayer patch containing ADH (PEO/PCL/PAC). Dashed lines correspond to the Korsmeyer–Peppas fitting. Values are presented as the mean ± SD (*n* = 3).

Figure 6 illustrates the cumulative release of the reservoir layer as a monolayer and within the multilayer patch. The release curves were fitted to the Korsmeyer–Peppas fitting model parameters (Table 4). Changes in the release rate among both systems were mainly attributed to the attenuated diffusion of the CPX through the adhesive layer in the multilayer patch. For both mono and multilayer, the values of the regression coefficient *(r^2^)* were high for the model used (Korsmeyer–Peppas), suggesting both a good fit and that more than one type of phenomenon of drug release was involved in the kinetics. In fact, the corresponding *n* values of the evaluated samples were below 0.5, which indeed indicated that the drug release appeared to follow pseudo-Fickian diffusional behavior.

As stated above, the multilayer patch led to a slower drug release rate compared to the reservoir monolayer. This observation could be mainly ascribed to the presence of a hydrophobic polymer (PCL) in the adhesive layer, which may hinder the CPX mobility across the adhesive layer, thus resulting in a slower drug release rate. This phenomenon is quantitatively characterized by the modeled parameter *K* (Korsmeyer–Peppas release rate), which is higher in the monolayer than in the multilayer approach. 

#### 3.2.3. Adhesion Test 

**Figure 7:**
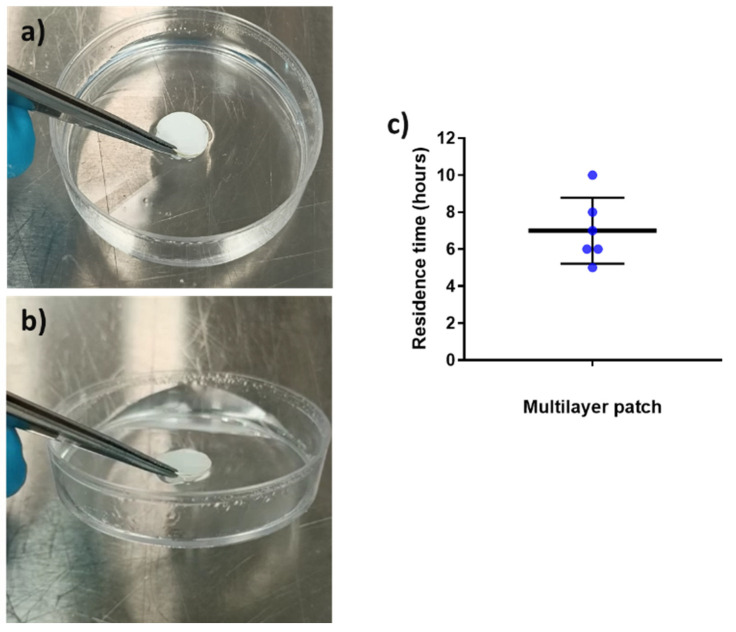
In vitro adhesion tests over time. (**a**) Multilayer placebo patch at time 30 min and (**b**) multilayer placebo patch at time 90 min, showing the lifting of the Petri dish. (**c**) In vivo mucoadhesive performance of the placebo multilayer patch, placed on the gingiva zone. Blue points indicate the individual results of all volunteers involved. The graph presents the mean ± SD (*n* = 6).

Mucoadhesion refers to the state in which material and the mucosa are attached together by means of interfacial forces [30]. This parameter is crucial to acknowledge the feasibility of a system to adequately adhere to the mucosa for a desired amount of time [54]. The adhesive formula presented in this work is a combination of hydrophilic (PEO) and hydrophobic (PCL and PAC) polymers since it is well reported in the bibliography that blends of polymers with opposite solubility can maintain layer consistency whilst improving the adhesive properties in the buccal environment [55,56]. The placebo multilayer structure was placed over the surface of a Petri dish containing a buffer solution, and its adhesion was checked after 30 and 90 min to assess the adhesive capabilities of the patch, as previously described in another study [11]. Figure 7a,b present the in vitro adhesion of the patch after 30 and 90 min, respectively. The patch remained strongly attached to the surface of the Petri dish after 30 and 90 min, to the point where it was possible to lift the Petri dish by pulling up the patch (see Figure 7b) due to the adhesion. In the adhesive layer (ADH), the presence of PEO, known to become an adhesive hydrogel in contact with water, and PAC with its ammonium-charged quaternary groups promoting ionic interactions with the negatively charged substrate [57,58,59], are thought to be the responsible materials to promote the observed strong adhesion. In light of these results, in vivo mucoadhesive study with healthy human volunteers was carried out (Figure 7c).

Placebo multilayer patches were placed in the gingiva zone of the mouth since previous studies had shown a better residence time in this area compared to the cheek or the tongue [28]. The patch residence time was ca. 7 h on average, with a variable distribution across the volunteers, probably due to the fact that no restrictions were given to the volunteers in terms of eating and drinking during the study (see Figure 7c). As shown in the in vitro adhesion study, the combination of the hydrophilic PEO and hydrophobic polymers PAC and PCL allowed the synergic prolonged adhesive behavior observed. The different solubility of the polymers that comprise the adhesive layer may have favored the consistency of the layer and, consequently, mucoadhesion. Additionally, as mentioned above, the cationic characteristics of PAC and the possible Van der Waals forces formed between the adhesive layer and the mucus area probably contributed to the adhesiveness [55,56,60,61,62]. In terms of the data on the controlled release mentioned above, it can be concluded that on average, the adhesive layer ADH adhered during the time required for a significant release of the CPX present in the patch.

#### 3.2.4. Antibacterial Activity

**Figure 8:**
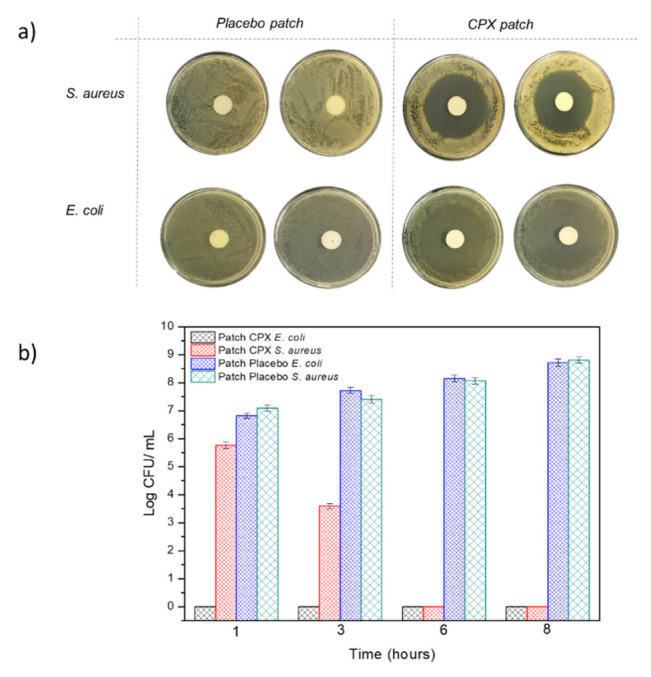
(**a**) Representative photographs of the growth inhibition against Escherichia coli (*E. coli*), and the Staphylococcus aureus (*S. aureus*) multilayer patch with and without CPX (n = 3) after 24 h. (**b**) Antibacterial reduction for Staphylococcus aureus (*S. aureus*) and Escherichia coli (*E. coli*) of the electrospun multilayer patches with CPX and without CPX (placebo) over a period of 1, 3, 6 and 8 h. Values are presented as the mean ± SD (*n* = 3).

The multilayer patch was also characterized in terms of its antibacterial activity against the Gram-positive and negative bacteria *E. coli* and *S. aureus* after 24 h. Figure 8a represents the inhibition zones presented by both the multilayer patches with CPX (CPX patch) and without CPX (placebo patch). The multilayer patches containing CPX showed a clear inhibition zone for both the Gram-positive and Gram-negative bacteria, with an inhibition halo of 5.92 ± 0.01 and 5.20 ± 0.01 cm, respectively, indicating the strong inhibition for both strains. These inhibition zones were not observed in the placebo multilayer patch, indicating the strength of CPX as an antimicrobial. The antibacterial activity was also measured at 1, 3, 6 and 8 h to simulate different patch residence times. Figure 8b shows the antimicrobial results of the multilayer patches with and without CPX. For the patches containing CPX, a high Log reduction was obtained after 1 h and also throughout the timeframe of the experiment for *E. coli*, whilst a lower inhibition was obtained against *S. aureus,* which growth was completely inhibited after 6 h. These results indicate that not only the CPX retains its properties after the electrospinning process but also that a strong antibacterial effect is observed even at low exposure times (less than 8 h). The effectiveness of the loaded electrospun CPX was also observed in other studies, with similar halo sizes to the ones reported here [34,49]. For instance, Li, H. et al. (2019) [34] carried out a study with CPX-loaded poly(vinylpyrrolidone) (PVP) and CPX-loaded ethyl cellulose (EC) fibers containing either 5 or 15% (*w*/*w*) of CPX with respect to the polymer concentration. The inhibition zones of the CPX-loaded PVP fibers at 5 and 15% CPX for *E. coli* and *S. aureus* after 24 h were 5.30 ± 0.61 and 5.71 ± 0.60 cm, whereas CPX-loaded EC fibers showed a smaller inhibition zone (4.29 ± 0.77–4.72 ± 0.23 cm), most likely due to the faster drug release rates of the PVP nanofibers compared to the EC material [34]. In another study reported by the same group, fibrous blends of poly (di(ethylene glycol) methyl ethermethacrylate) (PDEGMA) and poly(L-lactic acid-co-ε-caprolactone) (P(LLA-CL)) containing CPX showed inhibition zones for *E. coli* and *S. aureus* of 5.35 ± 0.51 cm and 5.21 ± 0.44 cm after 24 h, respectively. Similar values were obtained after 72 h, with halo sizes of approximately 5 cm [63]. These studies confirmed that CPX-loaded fibers could efficiently act as antimicrobial monolayer systems. Here, we have additionally shown that, even if enclosed in the middle layer of a multilayer patch, the CPX released from the fibers can gradually diffuse through the external layer, thus exerting an effective antimicrobial effect.

## 4. Conclusions

This work further demonstrates the feasibility of using electrospinning to generate fully functional drug delivery platforms, as is the case of a CPX-loaded multilayer patch. In this work, it was confirmed that using polymeric blends (in this case, PCL and PLA) and tuning the polymer ratio, it was possible to modulate the drug release rate. For demonstration purposes, the mixture 70PCL/3PLA was selected to generate the multilayer structure and was embedded between a backing and an adhesive layer to promote mucoadhesion and drug directionality. The release of the multilayer structure showed that the presence of an adhesive layer could also be used to further modulate the release rate of the API, acting as a rate-controlling membrane. 

The multilayer patch containing the electrospun blend 70PCL/30PLA interlayer was assessed in terms of adhesion and in vitro antimicrobial activity. The patch showed good adhesion in vitro as well as in vivo, with an average adhesion time in the gingiva zone of 7 h. The excellent antimicrobial properties of CPX were also corroborated in the multilayer structure across different time intervals. The results presented here illustrate the development of a model multilayer platform that can be potentially used as a buccal drug delivery system. Such drug delivery platforms could be further tuned with these or other polymers for the delivery of different BCS class II and IV APIs with use in multiple types of delivery strategies. Furthermore, electrospinning technology, now available at an industrial scale, could be quickly transferred to the pharmaceutical industry, opening new avenues for application in the pharmaceutical field.

## 5. Patents

The patch design developed in this research paper is based on a patent application PCT/ES2021/070294, in which some of the authors are inventors.

## Figures and Tables

**Table 1:** Composition of PCL-PLA polymer blend solutions, backing layer (BL) and adhesive layer (ADH).

Sample ID	Polymer Matrix and Ratio of the Polymer Blend (*w*/*w*)	Ratio Polymer/CPX (*w*/*w*)	Solvents and Ratio (*w*/*w*)
PCL	PCL	80/20	HFIP
80PCL/20PLA	PCL/PLA (80/20)	80/20	HFIP
70PCL/30PLA	PCL/PLA (70/30)	80/20	HFIP
60PCL/40PLA	PCL/PLA (60/40)	80/20	HFIP
50PCL/50PLA	PCL/PLA (50/50)	80/20	HFIP
20PCL/80PLA	PCL/PLA (20/80)	80/20	HFIP
PLA	PLA	80/20	HFIP
BL	PCL	-	Chloroform/Methanol (90/10)
ADH	PEO/PCL/PAC (70/17.5/12.5)	-	Chloroform/DMF (80/20)

**Table 2:** Electrospinning parameters of the PCL-PLA polymer blends solutions with and without CPX, backing layer (BL) and adhesive layer (ADH).

Sample ID	Flow-Rate (mL/h)	Voltage V+/V− (kV)	Needle-to Collector Distance (cm)
PCL	5	30/−25	15
80PCL/20PLA	10	15/−5	15
70PCL/30PLA	10	35/−25	15
60PCL/40PLA	10	35/−25	15
50PCL/50PLA	20	30/−15	15
20PCL/80PLA	10	25/−5	15
PLA	10	27/−20	15
BL	20	15/−2	15
ADH	15	25/−10	30

**Table 3:** Values of experimental CPX loadings in the different electrospun fibers as well as the yield of the process (%).

Sample ID	CPX Loading (%)	Yield (%)
PCL	19.9 ± 1.4	99.4 ± 1.4
80PCL/20PLA	19.6± 1.1	97.8 ± 0.5
70PCL/30PLA	19.5 ± 0.6	97.4 ± 2.8
60PCL/40PLA	19.4 ± 0.9	97.1 ± 1.4
50PCL/50PLA	19.8 ± 1.2	98.0 ± 1.5
20PCL/80PLA	19.7 ± 1.1	98.2 ± 1.1
PLA	19.3 ± 0.2	96.2 ± 0.9

**Table 4:** Model parameters of the CPX release profiles for the monolayer 70PCL/30PLA and multilayer patch obtained from the Korsmeyer–Peppas model.

Sample ID	*K*	*n*	*r^2^*
Monolayer	24.61	0.22	0.98
Multilayer	7.46	0.38	0.98
